# Mindfulness among lebanese university students and its indirect effect between mental health and wellbeing

**DOI:** 10.1186/s40359-023-01155-w

**Published:** 2023-04-13

**Authors:** Zeinab Bitar, Radosław Rogoza, Souheil Hallit, Sahar Obeid

**Affiliations:** 1 Faculty of Medicine, Paris-Saclay university, Le Kremlin-Bicêtre, France; 2 Cardinal Stefan Wyszyński University, Warsaw, Poland; 3 Social Innovation Chair, University of Lleida, Lleida, Spain; 4 School of Medicine and Medical Sciences, Holy Spirit University of Kaslik, P.O. Box 446, Jounieh, Lebanon; 5 Applied Science Research Center, Applied Science Private University, Amman, Jordan; 6 Research Department, Psychiatric Hospital of the Cross, Jal Eddib, Lebanon; 7 Social and Education Sciences Department, School of Arts and Sciences, Lebanese American University, Jbeil, Lebanon

**Keywords:** Mindfulness, Mental health, Depression, Anxiety, Wellbeing, University students, Lebanon

## Abstract

**Background:**

University students are a high-risk population for developing mental health issues. Mindfulness, the non-judgmental awareness of the present moment, has an effective role in numerous psychological contexts among students. However, no previous studies have investigated the association between mindfulness, mental health and wellbeing among Lebanese university students. Therefore, this study aimed to assess the mediating effect of mindfulness in the association between mental health and wellbeing in this population.

**Methods:**

This cross-sectional study enrolled 363 Lebanese university students recruited through convenience sampling (July-September 2021). The Wellbeing Index Scale, Lebanese Anxiety Scale, Patient Health Questionnaire and Freiburg Mindfulness Inventory were used to assess subjective well-being, anxiety, depression and mindfulness respectively.

**Results:**

Our findings showed that higher mindfulness (Beta = 0.18; p < 0.001) was significantly correlated with a higher wellbeing, whereas more depression (Beta=-0.36; p < 0.001) was significantly associated with a lower wellbeing. The results of the indirect effect analysis showed that mindfulness mediated the association between anxiety and wellbeing and between depression and wellbeing. Higher anxiety/depression were significantly associated with lower mindfulness and a lower wellbeing (direct effect). Moreover, higher mindfulness was significantly associated with a higher wellbeing.

**Conclusion:**

Mindfulness is associated with improved wellbeing and plays an indirect role between mental health issues and wellbeing. Our results suggest that mindfulness presents an adaptive approach and coping method associated with improved students’ wellbeing.

## Background

University students are considered a high-risk population for developing mental health issues, due to maladaptive health behaviors and stressing circumstances accompanying them during their academic programs [1, 2]. Students’ transition from high school to university is challenging, despite its important role in personal growth [3]. A survey enrolling 13,984 students around the world revealed that 35% reported at least one mental health problem [4]. According to the World Health Organization (WHO), mental health is a condition of wellbeing, through which an individual is able to cope with daily challenges, work professionally and fruitfully, and help the community [5].

Lebanon is lurching from a bad to worse situation, with the Lebanese people’s fragile mental health making them more sensitive to surrounding stressors [6–8]. Previous findings showed that 17.9% of Lebanese students present mild depression levels, 13.8% moderate depression and 1.7% severe depression [9]. Additionally, they indicated that 21.9% students had moderate anxiety, 6.3% severe anxiety and 2.3% extremely severe anxiety [9]. Depression and anxiety have been correlated with a negative impact on students’ academic achievement [10], low energy, social dysfunction and psychological distress [11].

Mental health problems can negatively affect a person’s wellbeing. Wellbeing is defined as a person’s evaluation of his/her life aspects (physically, mentally, socially and environmentally) [12]. A previous study revealed that a good wellbeing is associated with the absence of stress among students [13]. Authors explained that mood swings, as well as lack of sleep, energy, and appetite are correlated with higher risks of substance abuse, unemployment, and not achieving good grades, all of which leading to a poor wellbeing [13]. Moreover, low income among medical students can affect their mental health and consequently their wellbeing [14]; authors justified this association by the fact that low economic levels can lead to increased stress [15].

In order to protect students’ mental health, numerous preventative interventions were made; Mindfulness-Based Interventions (MBIs) were provided by a huge number of universities [16], as well as Mindfulness-Based Stress Reduction (MBSR) programs consisting of meditation, body scanning, breathing and yoga were adopted [17]. Mindfulness is defined as the non-judgmental awareness towards the present moment [18]; it consists of accepting and paying attention to whatever happens without any judgment or reaction as possible. Mindfulness represents a mode of positive and hardworking conscious thinking and a flexible mentality [17]. For instance, mindfulness skills allow people to get rid of various forms of maladaptive psychological conditions such as anxiety, depression, fear, anger, worry [19] and impulsivity [20].

Previous studies highlighted the effective role of mindfulness on numerous psychological contexts among students worldwide. A qualitative study revealed that students practicing mindfulness activities are more relaxed, less anxious and less stressed [19, 21]. Also, findings suggest that mindfulness represents a positive effect on mental health by the process of adaptive emotional regulation leading to reduced anxiety and depression [22]. Different studies conducted among athletes’ students showed that mindfulness may positively affect perceived performance by reducing depression, anxiety, and stress [23]; as well mindfulness is positively correlated with self-compassion and happiness [24]. Additionally, some studies revealed that mindfulness lead to efficient time management among students by controlling emotions and reducing worrying [25]. Moreover, a previous study highlighted the mediating effect of mindfulness between mental health and wellbeing among people; mindfulness mediated improvements in wellbeing and mental health [26–30]. Previous studies explained that mindfulness can enhance cognitive functions and therefore academic achievements [31]. Also, it improves detection, increases awareness and perceived positive emotions that will directly improve students’ mental health [32]. Consequently, when peoples’ stress is released and negative emotions are regulated, wellbeing improves [33].

### Rationale and aims of the study

The COVID-19 pandemic since its emergence and Lebanon’s financial crisis have shown to be detrimental on the psychological wellbeing of the undergraduate students by swelling the levels of stress, anxiety, and depression [6, 34]. Universities worldwide faced the risk of missing a whole academic year; therefore, many institutions in the world and in Lebanon switched to online learning [35], which has been associated with many maladaptive behaviors among Lebanese (i.e. phubbing) [36]. Therefore, the pandemic and the nation’s deteriorating socioeconomic conditions were having a significant cumulative impact on the mental health of its people, then followed by ethe Beirut explosion, which exacerbated Lebanon’s economic and political conditions. In particular, this had a significant impact on people’s mental health and living situations [37]. The current crisis is the worst in its history, leading to an increase in general psychological distress and suffering, affecting the wellbeing of the Lebanese population.

Given that consistent evidence suggests a positive association between poor mental health and wellbeing level, more efforts should be directed to explore modifiable factors (e.g., trait mindfulness) within this relationship [38]. More evidence supporting trait mindfulness as a mediator could help develop and implement mindfulness-based interventions aimed at enhancing this adaptive trait within low mindful young people, and subsequently decouple the relationship between poor mental health and low wellbeing.

Thus, this study aimed to assess the mediating effect of mindfulness in the association between mental health and wellbeing among Lebanese university students. We hypothesize that mindfulness would be directly associated with a higher wellbeing, while having an indirect role between anxiety/depression and wellbeing. In other words, lower anxiety and depression would be correlated with higher wellbeing, mediated by higher level of mindfulness.

## Methods

### Study design and participants

This cross-sectional study was carried out between July and September 2021. A total of 363 university students was recruited through convenience sampling in Lebanon’s governorates (Beirut, Mount Lebanon, South, North and Beqaa). Participants received the online link to the survey via the “WhatsApp” application, with an introduction containing the information form (purpose of the current study, anonymity, voluntariness of consent to research), followed by the questionnaire. Those university students were encouraged to forward the link to other students they know from the same or a different university, explaining the snowball technique. All university students over the age of 18 were eligible to participate; they responded willingly to the survey. The same methodology has been used in previous papers [39, 40].

### Measures

The Arabic self-administered questionnaire was anonymous, with closed-ended questions; it required approximately 20 min to be completed and consisted of different sections:

### Independent variables

The independents variables were sociodemographic characteristics (age, gender and marital status), physical activity and household crowding index. The physical activity index was calculated by multiplying the intensity by the frequency by the time of the physical activity [41]. The household crowding index reflects the socioeconomic status of the family, and was calculated by dividing the number of persons by the number of rooms in the house excluding the bathrooms and kitchen [42].

### Lebanese anxiety scale (LAS-10)

It consists of 10 items such as “I have intellectual problems (Difficulty in concentration, poor memory); I feel indecisive” assessing the severity of anxiety symptoms among Lebanese adults [43] and adolescents [44]. The first seven items are graded on a five-point Likert scale, not present to very severe [43]. The total score was obtained by adding up all responses, with higher scores indicating higher anxiety levels. The Cronbach’s alpha in this study was 0.89.

### Patient health questionnaire (PHQ-9)

It consists of 9 items assessing major depression symptoms such as “Little interest or pleasure in doing things; Being sad, depressed or hopeless”. The total score is obtained by adding up all responses; a total score ranging between [0–4] reflects no/minimal depression, [5–9] mild depression, [10–14] moderate depression, [15–19] moderately severe depression and [20–27] for severe depression [45]. The Arabic version of PHQ-9 was previously validated in Lebanon [46]. The Cronbach’s alpha in this study was 0.90.

### Dependent variable

#### The 5-item World Health Organization Well-Being Index (WHO-5)

It consists of 5 items such as “Over the last two weeks, I have felt cheerful and in good spirit”. These items assess subjective psychological well-being. Each item is scored on a 5-point Likert scale (0 = at none time to 5 = all of time). The total raw score, ranging from 0 to 25, is multiplied by 4 to obtain the final score, with 0 meaning the worst imaginable wellbeing and 100 reflecting the best imaginable wellbeing [47, 48]. The Arabic version of this instrument was validated in Lebanon [49]. The Cronbach’s alpha in this study was 0.95.

### Mediating variable

#### Freiburg Mindfulness Inventory (FMI)

FMI consists of 14 items to assess the person’s experience of mindfulness such as “I am open to the experience of the present moment; I am able to appreciate myself.” Each item is scored based on a 4-point Likert scale with 1 = rarely and 4 = always. Higher scores reflect more mindfulness; a score over 38 indicates effective mindful functioning [50]. The Arabic version of this scale has been validated in Lebanon [51]. The Cronbach’s alpha in this study was 0.91.

### Statistical analysis

A minimum of 316 university students was deemed necessary to have enough statistical power according to the G-power software (multiple regression; R^2^ deviation from zero) [52], based on a 5% risk of error, 80% power, f^2^ = 2.5% and 10 factors to be entered in the multivariable analysis.

SPSS software version 23 was used to conduct data analysis. We had no missing data since all questions were required in the Google form. Cronbach’s alpha values were recorded for reliability analysis of all scales. The wellbeing score was normally distributed, with its skewness and kurtosis ranged between − 1 and + 1 [53]. The Student t test was used to compare two means, whereas the Pearson correlation test was used to correlate two continuous variables. Cohen classified effect sizes as small (d = 0.2), medium (d = 0.5), and large (d ≥ 0.8) [54]. A forward linear regression was conducted to check for correlates of wellbeing. The PROCESS SPSS Macro version 3.4, model four [55] was used to test the indirect effect and calculate three pathways. Pathway A determined the regression coefficient for the effect of mental health issues (depression/anxiety) on mindfulness; Pathway B examined the association between mindfulness and wellbeing, and Pathway C’ estimated the direct effect of mental health issues on wellbeing. Pathway AB was used to calculate the indirect effect of depression/anxiety on wellbeing via mindfulness. An indirect effect was deemed significant if the bootstrapped 95% confidence intervals of the indirect pathway AB did not pass by zero [55]. Variables that showed a *p* < 0.25 in the bivariate analysis were taken as independent ones in the regression and indirect effect models. Significance was set at *p* < 0.05.

## Results

### Sociodemographic and other characteristics of the participants

The mean age of the participants was 22.65 ± 3.48 years, with 61.7% females. Other characteristics are summarized in Table 1.

**Table 1:** Sociodemographic and other characteristics of the participants (N = 363)

Variable	N (%)
**Sex**	
Male	139 (38.3%)
Female	224 (61.7%)
**Marital status**	
Single	343 (94.5%)
Married	20 (5.5%)
	**Mean ± SD (Min-Max)**
**Age (in years)**	22.65 ± 3.48 (18–37)
**Physical activity index**	27.94 ± 20.44 (1-100)
**Household crowding index**	1.01 ± 0.53 (0–4)
**Depression (PHQ-9)**	9.01 ± 6.34 (0–27)
**Anxiety (LAS-10)**	18.19 ± 8.22 (3–38)
**Wellbeing (WHO-5)**	13.50 ± 6.23 (0–25)
**Mindfulness (FMI)**	24.32 ± 8.56 (0–39)

Lebanese Anxiety Scale (LAS-10); Patient Health Questionnaire (PHQ-9); the 5-item World Health Organization Well-Being Index (WHO-5); Freiburg Mindfulness Inventory (FMI).

### Bivariate analysis

The bivariate analysis results are shown in Tables 2 and 3. A higher mean wellbeing score was seen in married students compared to single ones (16.80 vs. 13.31; p = 0.015). Older age (r = 0.130; p = 0.013) and higher mindfulness (r = 0.430; p < 0.001) were significantly associated with higher wellbeing, whereas higher anxiety (r=-0.407; p < 0.001) and depression (r=-0.488; p < 0.001) were significantly associated with lower wellbeing.

**Table 2:** Group comparison on wellbeing levels

	Mean ± SD	*p*	*t*	*df*	Effect size
**Sex**		0.276	1.092	361	0.120
Male	13.96 ± 5.81				
Female	13.22 ± 6.47				
**Marital status**		**0.015**	2.452	361	0.629
Single	13.31 ± 6.25				
Married	16.80 ± 4.73				

Numbers in bold indicate significant p-values

**Table 3:** Bivariate analysis of the continuous variables associated with wellbeing

	r	*p*
Age	0.130	**0.013**
Physical activity index	-0.001	0.981
Household crowding index	-0.046	0.384
Mindfulness total score	0.430	**< 0.001**
Anxiety	-0.407	**< 0.001**
Depression	-0.488	**< 0.001**

Numbers in bold indicate significant p-values; r = Pearson correlation coefficient.

### Multivariable analysis

A forward linear regression taking the wellbeing as the dependent variable, showed that higher mindfulness (Beta = 0.18; p < 0.001) was significantly associated with a higher wellbeing, whereas more depression (Beta=-0.31; p < 0.001) was significantly associated with a lower wellbeing (Table 4).

**Table 4:** Multivariable analysis: Linear regressions (using the ENTER model) taking the wellbeing score as the dependent variable

	Beta	β	*p*	95% CI
Model 1: Sociodemographic variables as independent variables.				
Age	0.15	0.08	0.199	-0.08; 0.38
Marital status (married vs. single)	2.07	0.08	0.239	-1.38; 5.53
Household crowding index	-0.30	-0.03	0.630	-1.52; 0.92
**Model 2: Sociodemographic variables and depression, anxiety and mindfulness as independent variables.**				
Age	0.03	0.02	0.768	-0.17; 0.22
Marital status (married vs. single)	0.78	0.03	0.605	-2.18; 3.74
Household crowding index	0.26	0.02	0.627	-0.79; 1.31
Anxiety	-0.05	-0.07	0.296	-0.15; 0.04
Depression	-0.31	-0.31	**< 0.001**	-0.43; -0.18
Mindfulness	0.18	0.25	**< 0.001**	0.10–0.26

*Reference group; Nagelkerke R^2^ values for model 1 = 2.2% and model 2 = 29.3%; Beta = unstandardized beta; β = standardized beta; CI = Confidence interval; numbers in bold indicate significant p-values.

### Indirect effect analysis

**Fig. 1:**
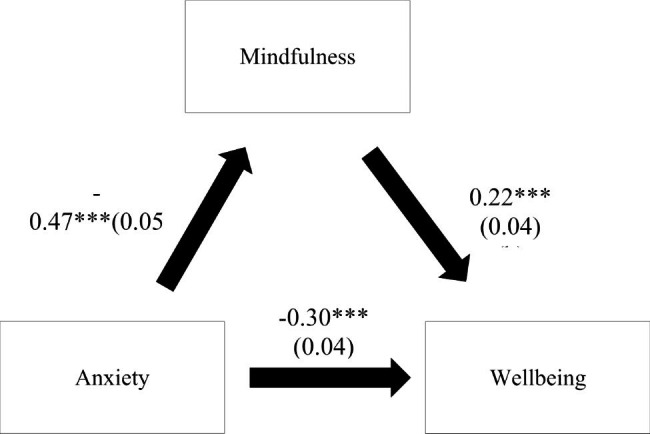
**(a) Relation between anxiety and mindfulness (R**^**2**^ **= 23.06%); (b) Relation between mindfulness and wellbeing (R**^**2**^ **= 24.08%); (c’) Relation between anxiety and wellbeing (R**^**2**^ **= 17.06%).** Numbers are displayed as regression coefficients (standard error). ***p < 0.001

**Fig. 2:**
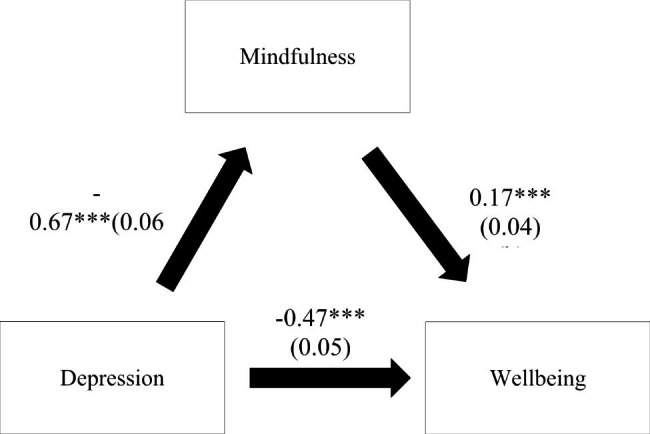
**(a) Relation between depression and mindfulness (R**^**2**^ **= 27.15%); (b) Relation between mindfulness and wellbeing (R**^**2**^ **= 28.46%); (c) Relation between depression and wellbeing (R**^**2**^ **= 24.27%).** Numbers are displayed as regression coefficients (standard error). ***p < 0.001

The results of the indirect effect analysis showed that mindfulness played an indirect role in the association between anxiety and wellbeing and between depression and wellbeing since the confidence interval of the indirect effect did not pass by zero (Table 5). Higher anxiety/depression were significantly associated with lower mindfulness and a lower wellbeing (direct effect). Moreover, higher mindfulness was significantly associated with a higher wellbeing (Figs. 1 and 2). These results are adjusted over the following sociodemographic variables: age, marital status and household crowding index.

**Table 5:** Indirect effect analyses results, taking anxiety/depression as independent variables, mindfulness as the mediator and wellbeing as the dependent variable

	Direct effect			Indirect effect		
	Beta	SE	*P*	Beta	Boot SE	Boot CI
Anxiety	-0.20	0.04	< 0.001	-0.10	0.02	-0.15- -0.06*
Depression	-0.35	0.05	< 0.001	-0.12	0.03	-0.18- -0.06*

* indicates significant indirect effect.

## Discussion

Our study investigated the associations between mental health issues (anxiety and depression) and wellbeing among university students in Lebanon, taking into consideration the indirect role of mindfulness in these associations. Our findings showed that mindfulness among Lebanese university students was positively associated with a higher wellbeing. Also, higher depression among those students was associated with lower wellbeing. However, the association between both anxiety/depression and between depression/wellbeing was mediated by mindfulness.

### Mindfulness and wellbeing

Findings in our study showed that mindfulness was positively associated with a higher wellbeing among Lebanese university students, in accordance with previous studies highlighting this positive association among the general healthy population [56, 57]. Literature explains that mindfulness presents an effective coping technique, enhancing wellbeing by decreasing depressive symptoms and anger [58]. Shapiro et al. [59] explained that the person practicing mindfulness perceives his thoughts non-judgmentally and therefore, will be more functionally adaptive. Additionally, the individual practicing mindfulness can feel high levels of self-confidence, optimism and can be more successful [60, 61]; these feelings will consequently lead to a better wellbeing. Moreover, a significant association has been found between mindfulness and positive affect [61], which has been known as an important factor enhancing wellbeing [62].

### Depression and wellbeing

Our results showed that more depression has been associated with a lower wellbeing among Lebanese students, corroborating the results of various studies among students worldwide [63–65]. This association can be explained by the personal and academic stressing circumstances among students, which enhance fear, ineptitude, anger and uselessness; consequently, they can lead to psychological and physical conditions affecting students’ wellbeing [66, 67].

### Mediating effect of mindfulness

The association between anxiety and wellbeing has been found to be mediated by mindfulness in our study, in accordance with previous findings [27, 28]. Penberthy et al. showed that college students attending meditation classes present increased mindfulness, mediating self-compassion and lower anxiety. They explained that mindfulness can improve educational performance by enhancing inter/intra- personal intelligence, and increasing concentration, memory and attention; therefore, students will acquire knowledge easily and be less worried about reaching high educational standards [28]. This association was also confirmed by previous findings showing that mindful students seeking perfect performance and high grades are less vulnerable to developing anxiety symptoms [68] and consequently, have a better wellbeing. Studies revealed that mindfulness-based interventions have been found to significantly reduce college students’ anxiety [69, 70], since mindful students are more optimistic, aware of the present moment and are not distracted by future or past stressing experiences [71–74].

Last but not least, mindfulness mediated the association between depression and wellbeing in our study. Gu et al. revealed that mindfulness mediates wellbeing and mental health outcomes [75]. Interestingly, this relationship could be explained by the significant correlation between mindfulness and having a purpose in life [76, 77]. The latter has been found to be negatively associated with many psychological illnesses such as depression [78, 79]. Mindfulness is also related to cognitive reappraisal, wisdom, appreciation of important things, and ignorance of traumatic experiences in life [80, 81], which may explain its mediating effect between depression and wellbeing.

### Clinical implications

Our study results suggest various practical implications for students’ development. Mindfulness presents an adaptive approach and a coping method associated with improved students’ wellbeing; thus, mindfulness-based interventions could be relevant during the student’s transition from high school to university.

According to the prevention paradox principle, there is growing interest in the functions of broad-based, school/university-integrated health promotion treatments that aim to target a variety of young resilience and protective variables [82]. One such resilience-building strategy that has been demonstrated in adolescent research studies to be effective for raising a variety of psychological adjustment and coping mechanisms as well as directly treating psychological distress, is mindfulness [83]. Research suggests that school- and university-based mindfulness resiliency techniques may be a cost-effective way to achieve government goals for juvenile mental health while also enhancing the wellbeing of parents, instructors, and students. Additionally, mounting research shows that mindfulness can enhance both general classroom behavior and student learning performance [84]. The fact that it appears mindfulness can also be delivered in an efficacious manner as an internet-mediated intervention further increases its appeal given the cost-effectiveness of this delivery mode [85].

We also suggest that mindfulness techniques (meditation, yoga, etc.) be taken into consideration and added to educational programs. Furthermore, parents might teach their kids about mindfulness since childhood and adolescence may be the ideal developmental period for introducing mindfulness in order to improve cognitive development (i.e. executive functions and self-regulation skills) [86]; hence, it can be adopted as a healthy behavior and an important self-care tool associated with improved mental health, which might consequently prevent future maladaptive behaviors such as substance abuse [86].

Last but not least, health care professionals can organize a clinical guide in which they identify how precisely mindfulness techniques are effective, their mode and the sufficient time of training to sustain healthy mental conditions.

### Limitations and strengths

Cross-sectional studies do not allow us to establish causal relationships. All responses obtained about mindfulness and psychological conditions were self-reported; responders may have over- or under-estimated some questions, which may result in an information bias. Additionally, other factors that might be related to mental health (year of study, major, type of university, type of faculty, practicing meditation/yoga/mindfulness) have not been assessed in the questionnaire, predisposing us to a confounding bias. A selection bias is also possible since the response rate is unknown and the fact that the sample was recruited conveniently; therefore, our results might not be generalizable to the whole population. Future studies taking these limitations into consideration and enrolling students randomly are needed.

## Conclusion

Our results revealed that mindfulness is associated with enhanced wellbeing and mediated the association between anxiety/depression and wellbeing. Research on mindfulness is still limited in educational fields; further research is needed to develop a solid background aiming at understanding how mindfulness works and specifying the effect of targeted mindfulness strategies among students. Despite the progress made in this field, it is likely that new patterns and techniques of mindfulness will continue to appear. This progress will allow students to attenuate psychological illnesses and help people live a grateful, happy and productive life.

## Data Availability

All data generated or analyzed during this study are not publicly available due the restrictions from the ethics committee (data are owned by a third-party organization). The dataset supporting the conclusions is available upon request to the corresponding authors.

## Abbreviations

COVID-19: Coronavirus Disease
FMI: Freiburg Mindfulness Inventory
LAS-10: Lebanese Anxiety Scale
MBIs: Mindfulness- based interventions
PHQ-9: Patient Health Questionnaire
WHO: World Health Organization
